# Predicting Exposure to Perfluorinated Alkyl Substances (PFAS) among US Infants

**DOI:** 10.3390/ijerph19148402

**Published:** 2022-07-09

**Authors:** Andrea B. Kirk, Kelsey Marie Plasse, Karli C. Kirk, Clyde F. Martin, Gamze Ozsoy

**Affiliations:** 1 Milken Institute School of Public Health, George Washington University, Washington, DC 20052, USA; 2 Department of Chemistry, Miami University, Oxford, OH 45056, USA; plassekm@miamioh.edu; 3 Department of Computer Science, University of Texas at Austin, Austin, TX 78712, USA; kk1377@protonmail.com; 4 Department of Mathematics and Statistics, Texas Tech University, Lubbock, TX 79409, USA; clyde.f.martin@ttu.edu; 5 Department of Pediatrics, Georgetown University School of Medicine, Washington, DC 20007, USA; gmzozsoy@gmail.com

**Keywords:** prenatal, PFAS, infant, exposure, PFOA, PFOS, PFDA, PFHxS, PFNA

## Abstract

PFASs have been detected in nearly every serum sample collected over the last two decades from US adults as part of the National Health and Nutrition Examination Survey (NHANES) and are commonly found in other data sets from around the world. However, less is known about infant PFAS exposures, primarily because the collection of infant serum samples is less common and frequently avoided. Cord blood samples are often preferred for chemical exposure assessments because this is thought to provide a good representation of infant serum concentrations, at least at the time of birth. In this paper, we will provide a statistical and probabilistic analysis of what can be expected for infants living in the US using NHANES from 2007 to 2008, which contains a rare subset of infant data. Regulatory efforts that require estimation of exposures among the very youth can be challenging, both because of a lack of data in general and because variability among this most vulnerable population can be uncertain. We report that US infant exposures are extremely common and that serum concentrations remain fairly constant, despite infant growth rates and relatively high caloric and fluid intake, with the possible exception of PFOS. Infant serum PFOS concentrations between months 1 and 3 are consistently higher than at less than one month, even though healthy infants at 1 and 2 months weigh more than they did at birth. This suggests that the babies are exposed to greater concentrations of PFOS after birth or that excretion kinetics differ for this PFAS.

## 1. Introduction

Per- and polyfluoroalkyl substances (PFASs) are a large group of synthetic chemicals with varying structures and unique properties. PFASs have a high thermal stability that has led to their use in firefighting foams. They also have nonstick properties that have been utilized in coatings of products that are oil and water-resistant such as non-stick cookware, carpets, textiles, mattresses, food packaging, stain removers, and soil repellents [1]. Consequently, PFAS have been used in a wide variety of commercial and industrial products since they first became available in the 1940s.

PFAS contain at least one fully fluorinated carbon. This is one of the strongest chemical bonds in nature. As a result, PFASs are persistent, slow to degrade and are often referred to as “forever chemicals”. PFASs bioaccumulate in wildlife [2] as well as in human tissue [3]. Data from the CDC [4] show that PFASs are present in the blood of 97% of Americans [5]. PFASs have been detected in brain [6], bone [3], placenta and fetal organs [7]. The PFASs with the highest concentrations reported in fetal organs were PFOS and then PFOA, PFNA/PFDA/PFUnA, with fetal organ concentrations similar to those found in placental tissue but lower than concentrations in maternal serum [8]. See Table 1 for abbreviations of PFAS.

There is rising concern about environmental toxicity and adverse human health effects of PFAS. As a result, government regulatory agencies are developing standards and guidelines for human and ecological PFAS exposure. In the US, different agencies and states have taken varied approaches towards risk assessment. Some, for example, estimate body burden at birth [9] while others assume exposure begins postnatally [10]. Different assessments may rely on the same or different animal study for points of departure or may differ in their approach to modelling a human equivalency dose or in estimating an expected safe level of exposure for humans. For chemicals such as PFASs, which can have half-lives of 5.3 years or longer [11], it will be important to model the onset of exposure and the time at which a serum steady state can be expected. Assessing fetal exposures and estimating PFAS body burdens accurately are important since this period is highly sensitive to disruption of key physiological processes that set the stage for the health of the future infant, child and adult.

In this paper, we examine serum concentrations of the 12 PFAS included in the NHANES 2007-8 analyses among infants under the age of one year. Sample sizes in this study are limited by the small number of infants tested for PFAS in the NHANES study. However, there is a well-developed science for the analysis of small samples. The theory of binary trials is the basis for most of the analysis presented here. A brief description of the underlying probability can be found in the Appendix A.

### What Is Known about the Effects of PFAS Exposures That Occur during Prenatal and Infant Life?

PFAS have a wide range of effects. In part, this is because they are different chemicals, with different molecular lengths and forms, and because they target different biochemical receptors. A single, individual PFAS may also interact with more than one molecular target [12]. The following are a few of the adverse effects observed in offspring either from direct interaction with fetal developmental processes or from interference with maternal or placental biology.

Neurodevelopment: Vuong et al. [13] found positive associations between maternal serum PFOS and PFNA concentrations and attention deficit hyperactivity disorder (ADHD) and between maternal serum perfluorohexanesulfonic acid (conjugate base of perfluorooctanesulfonate) (PFHxS) and externalizing and internalizing behaviors, but not for PFOA. Similar effects were found with greater externalizing behavior among both boys and girls for summed maternal PFAS (Perfluorooctanoic acid (PFOA), perfluorooctanesulfonic acid (PFOS), perfluorohexane sulfonate (PFHxS), perfluoroheptane sulfonate (PFHpS), perfluorononanoic acid (PFNA), perfluoro-n-decanoic (PFDA) and perfluoroundecanoic acid (PFUnDA) [14]. The authors noted that the study population was small and that their results should be interpreted cautiously. Other groups [15,16] have reported increased risks of autism spectrum disorder at age 3 with prenatal PFOA and PFNA exposures. Animal studies also support concerns for the neurodevelopmental risks posed by PFAS [17,18].

Metabolic health: There have been noted associations between cord blood PFAS and altered lipid profiles with particularly strong relationships between cord blood triglycerides and PFOA and PFHxS [19], between prenatal PFAS exposures and changes in cardio-metabolic profiles in infants [20] and additional associations between childhood serum PFAS and altered serum lipid profiles [21]. Additional studies show associations between prenatal PFAS exposures and increased adiposity during childhood [22].

Thyroid disruption: Relationships between thyroid function and PFAS in the mother and fetus have been reviewed [23,24]. Although results were often not completely consistent, an overall association between higher PFAS serum/plasma levels and a reduction in T4 levels and/or an increase in TSH levels has been described, especially for PFOS [25].

Immune function: Exposure to PFAS has been associated with reduced antibody responses to vaccines in young children. Exposure to PFAS during infancy resulted in lower antibody values for children vaccinated for diphtheria and tetanus when the children were evaluated at ages 5–7. Similarly suppressed antibody responses were also noted in adults [26]. While epidemiology studies are always vulnerable to confounding factors, immune response suppression is also observed in controlled animal experiments [27]. Others have examined maternal PFAS concentrations during gestation and frequencies of illness in young children, for example, respiratory tract and ear infections, varicella, etc. Higher maternal serum PFAS concentrations and presumably prenatal exposures are correlated with increased childhood infections [28].

Placental disruption: PFAS target important nuclear receptors (Peroxisome Proliferator Activating Receptors or PPARs) within the placenta [29,30], leaving the developing infant vulnerable to the effects of altered placental function in addition to targeting those same receptors within fetal tissues. A second potential mechanism for placental disruption is interference with angiogenesis [31], although this may be secondary to PFAS-induced PPAR activation. Interference with angiogenesis, and its effect on placental sufficiency, might be a cause of or a contributor to the lower birth weights observed with higher maternal serum concentrations of some PFAS [32], although effects seem to differ according to infant gender. An additional vulnerability is genetic polymorphisms, which may make the placenta of some women better able to transfer hazardous chemicals to the fetus [33]. More studies will need to be performed in this area that might explain differences in birthweight or other effects between male and female infants [34].

Continuing PFAS exposure during infancy: PFAS exposures continue postnatally, potentially through breastmilk [35], formula, water, other infant food sources, food packaging [36], consumer products or the home environment [37]. Young children, including infants, may be at greater risk of exposure through increased hand-to-mouth activity.

PFAS in Breastmilk: PFASs have been detected in human milk samples from at least the 1970s, although the profiles have changed over time [35]. Liu et al. [38] predicted that serum–breastmilk ratios and the rate of transfer of PFAS to milk are relatively low. Serrano et al. [39] reported, in a study of samples of donor suppled breast milk from Southern Spain, that a high percentage (36% to 100%) of the samples tested positive for various PFAS substances. A detailed discussion and analysis of PFAS in breastmilk was recently published by LaKind et al. [40].

NHANES collects limited information on breastfeeding: an infant would be classified as “breastfed” if he or she was reported as having received breastmilk on at least one of two days during the mother’s intake interview, but there is no indication of how frequently a breastfed child or what proportion of caloric or fluid intake could be attributed to this source. While exclusive breastfeeding is encouraged for the first six months of life and encouraged at least in part for the first year, only a minority of US infants reach these goals. About 46% of US infants are exclusively breastfed during the first three months of age and about 22% to 25% of US infants are exclusively breastfed during the first six months [41,42]. We could not attribute infant serum PFAS concentrations exclusively or predominantly to breastfeeding.

PFAS in infant foods: Few studies have been conducted on PFAS in food intended specifically for babies, although it has been detected in infant rice cereal [43]. More studies on PFAS in infant formula and baby food are needed, since either or both of these are consumed by nearly all US infants [44].

**This analysis was undertaken to address the following**:

- Determine probable incidence of infant exposure within the US.
- Determine if infant serum concentrations change during the first year of life.
- If infant serum concentrations change, are they associated with average ages of major infant milestones such as transition to solid foods?
- Raise awareness among policy makers and regulatory scientists of infant serum PFAS concentrations and infant and fetal PFAS exposures in general.

This analysis is important because the details of infant exposures and serum concentrations are not well understood. Understanding changes among infant exposures or serum PFAS concentrations is important for identifying key dietary or behavioral changes that might indicate periods of increased risk. Lastly, it is important to understand serum concentrations among very young children as the average serum concentrations of a total population might be quite different from this particularly vulnerable sub-population. We recognize that breast milk, cord blood, fetal tissues and placental analyses are all good tools for evaluating fetal and infant exposures to environmental chemicals. We chose to focus on infant serum concentrations for this analysis because serum concentrations and estimates of serum concentrations are so important in regulatory decision making. Chemical risk assessors correlate serum concentrations with adverse outcomes and use these as the basis for determining how much of a chemical should be allowed in drinking water or other potential sources of human exposures.

## 2. Materials and Methods

The data for this study are publicly available and taken from the 2007–2008 NHANES [4]. Survey design details and methodology can be accessed on the NHANES website (https://www.cdc.gov/nchs/nhanes/index.htm (accessed on 15 January 2022)). In summary, NHANES is an ongoing survey of the non-institutionalized US population that utilizes a stratified, multistage probability sampling protocol. After acquiring written informed consent, physical assessments, examinations and laboratory measures are completed in mobile examination centers (MEC). Basic statistics and information for infants under the age of 1 (*n* = 93) are shown in Table 1. The number of infants in each age group (by month) is small (Table 2, and we will use the percentage of infants that have serum PFAS above the limit of detection (percent positive) (Table 3) to make estimates of the percentage that will test positive in the general population

We also examined exposures among pregnant and non-pregnant women represented by this data set. Nearly all women had detectable PFAS in their serum. Fifty-six pregnant women between the ages of 20 and 44 participated in that year’s NHANES survey. Of these, 12 had their blood samples tested for PFAS. All had concentrations above the limits of detection (LOD) for at least one PFAS. It is important to keep in mind that analytical methods have improved, and a non-detect could be a “detect” if the sample had been analyzed a year or so later. For example, the LODs for MeFOSAA and PFOS for the 2017–2018 NHANES survey was 0.1 ng/mL, while in 2007–2008, the LODs were 0.2 ng/mL

Using the data from Table 3, we are able to provide predictions on the number of infants that will have detectable PFAS in the general population. The numbers are calculated using the mean and standard deviation based on the probability distribution of a binary trial. Recall that a binary trail arises when we have an unknown population that is labeled either red or green. We draw from the population and draw n reds and m greens. With this information, we would like to estimate the true percentage of greens in the population. We would guess that the true percentage is given by b/(r + g), which is the percentage drawn, but we can almost be sure that this is not correct. This situation has been studied extensively and we can have some assurance about the mean and standard deviation from the theory of binary trials. This is further explained in the Appendix A.

Note that even though, in some cases, 100% of serum samples tested above LOD, it is to be expected that there will be infants that test below it. Likewise in cases where only 1 or 2 tested positive, it is to be expected that there will be more in the general population (Table 2).

**Data summary**. The 2007–2008 NHANES data set contains information for 10,149 subjects, of which 454 were under the age of 1. Of these, 101 were tested for serum PFAS (Table 3). Samples were analyzed by tandem mass-spectrometry using the method developed by Kuklenyik et al. in 2006 [45] and were reported in ng/mL. Limits of detection varied (Table 1). NHANES uses a complex strategy to ensure that sampling is representative of the US population. However, no explicit target samples sizes were made for infants; thus, the population of infants surveyed may not truly reflect the US infant population. Subsamples of 1/3 of available samples were made for subjects aged 12 and over. We were unable to determine how the subsample of infants (23% of the total infant cohort) was selected for serum PFAS testing.

## 3. Results

The first thing to note from Table 4, representing PFOA, PFNA, PFHxS, PFDA and N-MeFOSAA, is that the regression lines are very flat. This indicates that there is little change in serum concentrations from birth to age 1, and that there is no support, at least in this data set, for the hypothesis that changes in infant serum PFAS are associated with behavioral or dietary changes. Additional data tables (Table 5, Table 6, Table 7, Table 8 and Table 9) are provided because the data are not readily visible on the NHANES site and require some manipulation. They can be observed under their respective subheadings. The one PFAS that appears to differ from this trend is PFOS, which is represented in Table 10.

### 3.1. PFOA

Here again, we use results from the theory of binary trials, which is standard when studying small samples. Confidence intervals are large because of the small sample size. In the case of PFOA and the newborns, we have *n* = 8, and they all tested positive. We want to know the percentage of the general population of newborns that will test positive at least half of the time; thus, we use equation p^8^ = 0.5. From this, 8 log(*p*) = log(0.5) such that log(*p*) = log(0.5)/8 = −0.0866; hence, *p* = 0.917. Thus, there is a very high probability that the general population of US infants would also have detectable concentrations of PFAS in blood. We can provide a confidence interval for the probability in the general infant population by solving equations *p*^8^ = a and *p*^8^ = 1−a. For the 90% confidence, we used a = 0.5, and we have (0.6877, 0.994); thus, we are confident at the 90% level that the true percentage of babies with detectable PFAS in serum with the detection limit available at the time lies within this interval (Table 5).

### 3.2. PFNA, PFHxS, PFDA and N-MeFOSAA

As with PFOA, the data for PFNA, PFHxS and N-MeFOSAA are very flat, with slopes −0.0651, 0.002, −0.009 and −009, respectively (Table 6, Table 7, Table 8 and Table 9). It appears very probable that the babies were exposed in utero and continued exposure postnatally. At first glance, it may seem that if serum concentrations are stable, then further exposure is minimal, given the long half-lives expected for PFAS. However, it is important to consider the rapid rate of infant growth. If serum concentrations remain stable, while body mass increases, serum concentrations remained the same over time, but the source of exposure continues. On average, infants double in weight between birth and 3–4 months of age [46].

### 3.3. PFOS: The Case of Perfluorooctane Sulfonic Acid

This chemical, PFOS, appears to be different than the other three PFAS selected for analysis. Note that the slope of the medians over age is fairly large, −0.575 (Table 10), at least compared to the others.

The differences are most clearly observed in the data for three-month-old infants. The value of 99 ng/mL for a three-month old infant is an extreme outlier, with more than 3 standard deviations from the mean (mean = 20, Stdev = 25). The data for months one through four are consistently higher than the data for month 0, even though healthy infants at these ages would weigh more than they did at birth. This suggests that the babies are exposed to PFOS after birth to a greater degree than other PFAS and might reflect greater lactational transfer.

## 4. Discussion

As outlined in the introduction, there are many potential sources of PFAS exposure for infants: breast milk [47], water, formula [48], the household or childcare environment and/or baby food [44]. It will be important to understand these and also other factors, such as the effects of previous pregnancies, breastfeeding history and use of birth control [49] or other hormonal treatments or exposures, including endocrine disruptors, that may influence maternal PFAS excretion or kinetics before, during or after pregnancy. PFASs have been detected in cord blood and placenta [50]; thus, it is to be expected that there has been exposure prior to birth. Additional research is required to determine how these measures relate to total infant body burden at birth, but it appears that PFASs have different maternal to fetus transfer rates with significant differences between the profiles of maternal serum and cord blood [51].

A large study of cord blood samples from women who delivered infants in the Baltimore, MD, USA, area [52], around the same time as the NHANES 2007–2008 data were collected and analyzed by the same method [45], showed median cord blood serum for PFOA at 1.6 ng/L (range = 0.3 to 7.1 ng/mL) and 5.0 ng/L (range < LOD to 34.8 ng/mL) for PFOS. These cord blood values are lower than any of the medians for infant serum from the NHANES cohort (Table 5 and Table 10). Median PFOS serum values, reported here, were higher at 8.1 ng/L (range 5.0 to 31.5) even for the very youngest infants. At less than one month of age, the median infant serum value for PFOA was 2.65 (range 1.6 to 4.9). These are, of course, different cohorts, with NHANES designed to represent the US population in general, and the Baltimore study surveyed women who gave birth at a specific hospital. It may be that infant serum PFAS concentrations (or at least PFOA and PFAS) exceed those of cord blood. If so, we would not be able to use cord blood as a direct surrogate for infant serum in risk assessments. A better potential comparator would be the NHANES 2007-8 data on pregnant women. Only 10 of the pregnant women included in that year’s data set included serum PFAS values. The median serum concentration for these women was 3.4 ng/mL (range 1.1 to 8.3) for PFOA compared to 2.65 (range 1.6 to 4.9) for unrelated neonates. For PFOS, the median serum value for pregnant women was 11.4 (range 2.7 to 39.8) and 8.1 (range 5.0 to 31.5) for unrelated neonates. These are quite similar; however, it would be beneficial to obtain more information so that we can know how well maternal serum PFAS concentrations might predict those of infants. Information on stage of pregnancy was, unfortunately, not available, although with such small numbers of women, it would have been impossible to draw general conclusions about temporality and serum PFAS concentrations among this population.

## 5. Conclusions

For chemicals such as PFAS, which can have half-lives of 5.3 years or longer, it will be important to model the time at which a serum steady state can be expected. As nearly all women sampled have serum in their blood streams and examining the supporting data from studies of placental tissue and cord blood, it is more than reasonable to assume that nearly all pregnant women have detectable PFAS in their serum and that the life-time exposure of offspring to these chemicals begins in utero. Assessing fetal exposures accurately is important as this period is highly sensitive to disruption of key physiological processes that set the stage for the health of the future infant, child and adult. We found, with the possible exception of PFOS, that infant serum concentrations remain fairly stable from very early life (less than one month of age) to at least age one.

## 6. Study Limitations

This study has some of limitations that leave a number of unanswered questions:

- Data for infant exposures were not available for 2009–2010, 2011–2012, 2013–2014, 2015–2016, 2017–2018 or, as of yet, later NHANES, although we are hopeful that data, from some source, will be released in the future. The lack of infant serum data for PFAS dating later than 2007–2008 is a limitation for this study. More recent data would have allowed for analysis of changes in serum concentrations and PFAS ratios over time.
- The numbers of infants sampled who also had serum PFAS analyzed was small (93), and the numbers of infants at any of the 12 age groups (0–11 months) was smaller still. Larger numbers would have allowed for finer estimates.

## Figures and Tables

**Table 1:** Serum PFAS data for infants <1 year of age.

PFAS Abbreviation	Chemical Name	LOD ng/mL	% > LOD	Mean ng/mL	Median ng/mL	95th Percentile
**2-(N-ethyl-PFOSA) acetate**	2-(N-Ethyl-perfluorooctane sulfonamido) acetic acid	0.2	2	<LOD	<LOD	<LOD
**PFDA**	Pefluorodecanoic acid	0.2	67	0.41	0.3	0.9
**PFOA**	Perfluorooctanoic acid	0.1	100	4.01	3.5	7
**PFOS**	Perfluorooctane sulfonic acid	0.2	100	16.8	11	36.4
**PFHxS**	Perfluorohexane sulfonic acid	0.1	100	3.07	1.6	4.2
**N-MeFOSAA**	2-(N-Methyl-perfluorooctane sulfonamido) acetic acid	0.2	62	0.46	0.2	0.5
**PFBS**	Perfluorobutane sulfonic acid	0.1	0	<LOD	<LOD	<LOD
**PFHPA**	Perfluoroheptanoic acid	0.4	8	0.30	<LOD	<LOD
**PFNA**	Perfluorononanoic acid		89	1.47	1.1	2.38
**PFOSA**	Perfluorooctane sulfonamide		1	0.07	<LOD	<LOD
**PFUnDA**	Perfluoroundecanoic acid		31	0.28	<LOD	0.07
**PFDoDA**	Perfluorododecanoic acid		1	0.14	<LOD	0.14

**Table 2:** Subject Age and Gender.

**Age in Months**	<1	1	2	3	4	5	6	7	8	9	10	11
**Males (46)**	3	5	4	5	4	3	4	1	2	6	7	2
**Females (55)**	5	4	3	8	4	3	6	4	5	6	2	5
**Total number of infants (101)**	8	9	7	13	8	6	10	5	7	12	9	7

**Table 3:** Expected percentages in the general US infant population.

PFAS	Number Positive	Expected Percent	Conf. Interval
**2-(N-ethyl-PFOSA) acetate**	2	3%	(0.2, 6)
**PFDA**	67	66%	(58, 74)
**PFOA**	101	98%	(96, 100)
**PFOS**	101	98%	(96, 100)
**PFhxS**	101	98%	(96, 100)
**N-MeFOSAA**	62	61%	(53, 69)
**PFBS**	0	1%	(0, 2.5)
**PFHPA**	8	9%	(4, 13)
**PFNA**	89	87%	(82, 93)
**PFOSA**	1	1.9%	(0, 4)
**PFUnDA**	31	31%	(24, 38)
**PFDoDA**	1	1.9%	(0, 4)

**Table 4:** Summarized data from the 2007–2008 NHANES data set for the five PFAS most commonly detected in infant serum. PFAS.

*N*=	Age in Months	Median PFDA (ng/mL)	Median PFOA (ng/mL)	Median PFOS (ng/mL)	Median PFHxS (ng/mL)	Median N-MeFOSAA (ng/mL)
**8**	<1	0.17	2.65	8.1	1.1	0.35
**9**	1	0.5	3.7	33.6	1.7	0.2
**7**	2	0.7	3.3	19.5	1.9	0.3
**13**	3	0.3	3.9	11	1.5	0.3
**8**	4	0.14	3.8	11.1	2	0.2
**6**	5	0.35	4.8	15.75	2.15	0.45
**10**	6	0.25	3.75	8.95	1.3	0.25
**5**	7	0.3	3.4	17.9	1.9	0.12
**7**	8	0.25	3.55	13.1	1.75	0.21
**12**	9	0.3	3.9	11.95	2.15	0.3
**9**	10	0.2	4.3	14	1.4	0.3
**7**	11	0.2	3.3	11.8	1.4	0.12
**Slope**		−0.01762	0.04458	−0.575	0.008566	−0.00881
**intercept**		0.401923	3.450641	17.89167	1.640385	0.306795

**Table 5:** Individual and median PFOA concentrations, slope and intercept.

Perfluorooctanoic Acid (PFOA) in µg/mL												
Age (Months)	0	1	2	3	4	5	6	7	8	9	10	11
**Median**	**2.65**	**3.7**	**3.3**	**3.9**	**3.8**	**4.8**	**3.75**	**3.4**	**3.55**	**3.9**	**4.3**	**3.3**
	1.6	3.6	4.6	4.6	4.9	6.3	4.3	3.4	4.7	6	7.5	7
	4.9	3.7	9.5	9.5	4.6	5.1	7.2	2.9	4.8	8.5	5.3	6.2
	4.8	9.7	2.5	2.5	3.8	4.8	4.1	6.7	2.3	1.7	5.6	3.8
	3.5	8.9	6.8	6.8	2.7	4.8	4.2	3.3	2.4	4.2	4.3	2.5
	2.5	10.2	3.3	3.3	6.8	3	2.8	3.6	3.3	6.4	6	3.3
	2.2	2.9	3	3	2.3	3.1	3.4		3.8	3.4	2.1	2.7
	2.2	2.5	0.4	0.4	4.8		2.1			3.7	2.4	1.8
	2.8	4.3	4.6		2.9		4.6			4.1	4	
		2.5			2.5		2.8			4.1	3.2	
							0.8			3.7		
										2.4		
										0.3		
**Slope**	**0.04458**											
**Intercept**	**3.450641**											

**Table 6:** Individual and median PFNA concentrations, slope and intercept.

Perfluorononanoic Acid (PFNA) in ng/mL												
Age (Months)	0	1	2	3	4	5	6	7	8	9	10	11
**Median**	**0.984**	**1.886**	**1.968**	**1.394**	**0.82**	**1.476**	**0.984**	**1.394**	**1.066**	**0.14**	**0.984**	**1.23**
	1.558	2.296	2.542	10.988	1.558	2.132	1.394	1.394	4.346	0.14	2.706	1.722
	1.066	4.838	2.05	1.476	1.722	1.558	2.378	2.378	1.394	0.14	0.984	2.624
	0.492	2.214	2.132	3.772	0.82	1.804	0.984	1.312	0.902	0.2	1.64	1.23
	1.312	1.886	1.968	1.394	0.82	1.394	0.656	0.82	1.066	0.14	1.23	0.738
	0.656	3.198	1.804	0.82	1.066	0.41	0.902	1.558	1.066	0.3	0.738	1.312
	0.984	1.64	0.656	1.066	0.656	0.656	1.066		0.656	0.3	1.23	0.574
	0.984	0.984	0.738	0.902	0.984		0.82			0.14	0.902	0.902
	0.656	0.574		1.968	0.738		1.23			0.2	0.492	
		1.476		1.804	0.574		0.984			0.14	0.82	
				1.476			0.492			0.2		
				0.738						0.14		
				0.574						0.14		
				0.41								
**Slope**	**−0.0651**											
**Intercept**	**1.551872**											

**Table 7:** Individual and median PFHxS concentrations, slope and intercept.

Perfluorohexane Sulfonic Acid() in ng/mL												
Age (Months)	0	1	2	3	4	5	6	7	8	9	10	11
**Median**	**1.1**	**1.7**	**1.9**	**1.5**	**2**	**2.15**	**1.3**	**1.9**	**1.75**	**2.15**	**1.4**	**1.4**
	0.9	1.6	17.2	12.6	4.4	4.2	2.2	3.1	1.2	9.9	5.2	12.7
	6.3	1.7	2.7	1.2	3.9	14.1	1.2	1.9	2.7	2.4	9.7	1.7
	2.7	4.9	3.7	1.5	2.3	1.2	5.7	12.9	0.8	3.3	5.4	0.7
	1.3	3	1.9	2	2	3.1	5	1.2	1	5.3	1.1	3.6
	0.5	3.6	0.3	1.6	0.9	1	1.4	0.5	2.3	2.7	7.5	1
	11	1.9	1.2	3.8	1.6	0.7	3.5		3.6	0.8	1.1	1.4
	0.7	1.3	0.1	1.1	4.1		0.4			4	0.7	0.6
	0.7	1.2		3.3	1.4		0.8			0.7	1.4	
		1.1		1.1	0.9		0.7			1.9	0.9	
				1.3			0.3			0.7		
				0.9						1.4		
				2.3						0.1		
				0.6								
**Slope**	**0.008566**											
**Intercept**	**1.640385**											

**Table 8:** Individual and median PFDA concentrations, slope and intercept.

Perfluorodecanoic Acid (PFDA) in ng/mL												
Age	0	1	2	3	4	5	6	7	8	9	10	11
**Median**	**1.3**	**1.893**	**1.802**	**1.579**	**1.763**	**1.4**	**1.479**	**2.3**	**1.397**	**1.45**	**1.67**	**1.7**
	0.6	0.2	2.6	1.2	2.2	0.4	0.3	0.4	0.9	0.14	0.3	0.4
	0.2	0.14	1	0.7	1.3	0.3	1.1	0.7	0.3	2.3	0.3	0.7
	0.14	0.14	0.5	0.7	0.9	0.2	0.14	0.14	0.14	0.4	0.7	0.2
	0.14	0.14	0.5	0.7	0.2	0.14	0.14	0.14	0.2	0.14	0.3	0.3
	0.14	0.2	0.7	1	0.2	0.3	0.2	0.3	0.3	0.5	0.14	0.2
	0.2	0.2	0.3	0.14	0.14	0.14	0.2		0.14	0.5	0.2	0.2
	0.2	0.14	0.14	0.4	0.3	0.14	0.4			0.14	0.14	
	0.14		0.14		0.4	0.14	0.5			0.5	0.14	
			0.5		0.7	0.14	0.3			0.3	0.14	
					0.4		0.14			0.3		
					0.2					0.14		
					0.14					0.14		
					0.14							
**Slope**	**0.002479**											
**Intercept**	**1.630782**											

**Table 9:** N-MeFOSAA Individual and median PFOA concentrations, slope and intercept.

2-(N-methyl-PFOSA) Acetate in ng/mL												
Age (Months)	0	1	2	3	4	5	6	7	8	9	10	11
**Median**	**0.35**	**0.2**	**0.3**	**0.3**	**0.2**	**0.45**	**0.25**	**0.12**	**0.21**	**0.3**	**0.3**	**0.12**
	0.3	1.7	0.3	0.5	0.3	0.8	1.4	0.12	0.12	0.5	5.8	0.12
	0.6	0.12	0.4	0.12	0.12	0.12	0.3	0.12	0.12	0.6	0.7	0.8
	0.3	0.2	0.4	0.12	0.4	0.3	0.12	2	0.3	0.12	0.12	0.2
	0.2	0.7	0.2	0.3	0.2	0.6	0.12	0.12	0.8	0.3	0.3	0.3
	0.4	3.3	0.12	0.8	1.5	0.2	0.2	0.4	0.3	0.4	0.12	0.12
	0.8	0.12	0.3	0.7	0.12	0.8	0.4		0.12	2	1.5	0.12
	0.12	0.2	0.2	0.12	0.12		0.3			0.12	0.12	0.12
	0.4	0.12		0.6	0.3		0.3			0.12	0.3	
		0.12		0.12	0.12		0.12			0.12	0.3	
				0.2			0.12			0.3		
				0.6						0.3		
				0.4						0.12		
				0.12								
**Slope**	**−0.0088112**											
**Intercept**	**0.30679487**											

**Table 10:** PFOS Individual and median concentrations, slope and intercept.

Perfluorooctane Sulfonic Acid (PFOS) in ng/mL												
Age (Months)	0	1	2	3	4	5	6	7	8	9	10	11
**Median**	**8.1**	**33.6**	**19.5**	**11**	**11.1**	**15.75**	**8.95**	**17.9**	**13.1**	**11.95**	**14**	**11.8**
	31.5	57.2	57.3	99.2	38.9	29.8	30.5	21.4	38.4	29	45.2	35.3
	15.5	38.6	32.4	34.5	17	28.5	19.4	18.5	25.7	20.1	23.2	19.3
	11.3	38.5	30.8	23.9	15.6	18.5	11.2	17.9	14.6	14.5	19.3	13.9
	8.3	36.4	19.5	20.4	13.7	13	10.7	9.4	11.6	14	15.2	11.8
	7.9	33.6	13.4	16	11.1	8.7	9.2	6.1	10.3	12	14	6.3
	6.1	10.9	6	15.5	10.9	2.5	8.7		7.4	12	8.3	5
	5	9.5	1.7	11	10.8		8			11.9	6.7	4.9
	4.8	7.2		10.5	5.3		6.5			8.2	5.5	
		4		10.5	2.8		5.7			8	4.6	
				10.1			3.1			6.9		
				5.5						5.1		
				3.5						1.5		
				2.7								
**Slope**	**−0.575**											
**Intercept**	**17.89167**											

## Data Availability

Data used in this study are available from the US Centers for Disease Control’s National Health and Nutrition Examination Survey. https://www.cdc.gov/nchs/nhanes/index.htm (accessed on 15 January 2022).

## Appendix A. Analysis of Binary Trials Related to Infant Exposure

Suppose that, from a population consisting of subjects who are either positive or negative, we have drawn a sample of N subjects and n of them have tested positive and m of whom have tested negative. We would like to know the true percentage of positives in the general population. Clearly, it is impossible to know the true proportion exactly without testing every single subject. We can, however, provide the best estimates.

Suppose the true probability is *p*; then, the probability of drawing *m* positives in a sample of size *n* is given by the following:

(A1) $$C_{n,m}p^{n}{\left(1-p\right)}^{n-m}.$$

here

(A2) $$C_{n,m}=\frac{\left(n+m\right)!}{n!m!}$$

and it is called the combinatorial symbol. The probability of drawing a positive is *p*; hence, the probability of drawing n positives is $p^{n}$ and the probability of drawing a negative is (1 − *p*). Hence, the probability of drawing m negatives is ${\left(1-p\right)}^{m}$. However, there are $C_{n,m}$ different orders in which they could have been drawn; hence, the formula is as follows.

Let the following be the case:

(A3) $$F\left(p\right)=C_{n,m}p^{n}{\left(1-p\right)}^{m}$$

and note that F is defined for all p∈[0,1] and we have F(0)=F(1)=0. We will make F(p) into a probability distribution function by finding a constant K such that the following is the case.

(A4) $$K\int_{0}^{1}F\left(p\right)dp=1$$

Using the fact that we have the following.

(A5) $$C_{n,m}\int_{0}^{1}x^{n}{\left(1-x\right)}^{m}dx=\frac{1}{n+m+1}$$

We have K=n+m+1. Thus, the distribution function is as follows.

(A6) $$G\left(p\right)=\left(m+n+1\right)C_{n,m}p^{n}{\left(1-p\right)}^{m}.$$

Now, given a probability distribution function P(x), the expected value, E(P), is given by the following.

(A7) $$\int_{0}^{1}xP\left(x\right)dx=E\left(P\right).$$

Now, we can calculate the expected value. First, we note that by a bit of manipulation:

(A8) $$C_{n,m}\left(n+m+1\right)=\left(n+1\right)C_{n+1,m}$$

we can obtain the following.

(A9) $$\begin{array}{rrr} \int_{0}^{1}pG\left(p\right)dp & = & \left(n+m+1\right)C_{n,m}\int_{0}^{1}p^{n+1}p^{m}dp\\ & = & \left(n+1\right)C_{n+1,m}\int_{0}^{1}p^{n+1}{\left(1-p\right)}^{m}dp\\ & = & \frac{n+1}{n+m+2} \end{array}$$

We thus have the following:.

Fact 1: Given that a sample of *n* positives and *m* negatives has been drawn from a population. The expected value and mean value of the fraction of positives in the general population are as follows.

(A10) $$\frac{n+1}{n+m+2}$$

We now want to establish a confidence interval about this calculated mean value. Say at the 90% level, we want to construct an interval that includes 90% of the area under the curve given by G(p). The usual way to obtain this is to solve the following two equations for *t* and *s*.

(A11) $$\begin{array}{rrr} \overset{t}{\underset{0}{\int }}G\left(p\right)dp & = & 0.05\\ \overset{s}{\underset{0}{\int }}G\left(p\right)dp & = & 0.95. \end{array}$$

In general, these equations are very difficult to solve and one must revert to approximation methods.

Fact 2: Given that a sample of n positives and m negatives has been drawn from a population, the 90% confidence interval for the mean is given by (t,s) where *t* and *s* are calculated as below.

**Example** **A1.**
*n positive and 0 negative.*

We have drawn *n* subjects and all *n* tested positive. Then, we have $C_{n,0}p^{n}$ as our function and $C_{n,0}=n$. Thus, using Fact 1, the expected value of the true probability is $\frac{n+1}{n+2}$ which proceeds very close to 1 for large samples. To calculate the confidence interval, we integrate the following.

(A12) $$\int_{0}^{t}(n+1)p^{n}dp=p^{n+1}$$

Thus, we solve $p^{n+1}=0.05$ and $p^{n+1}=0.95.$ When *n* is, for example, 10, we have mean= 11/12= 0.92, and the 90% confidence interval is (0.76, 0.99).

**Example** **A2.**
*n positive and 1 negative.*

We have drawn *n* + 1 subjects and *n* tested positive and 1 tested negative. $C_{n,1}=n+1$ and we search for a constant *K* so that the following integral is obtained.

(A13) $$K\int_{0}^{1}p^{n}\left(1-p\right)dp=K\frac{1}{\left(n+1\right)\left(n+2\right)}=1$$

Therefore, the probability distribution function is $\left(n+1\right)\left(n+2\right)p^{n}\left(1-p\right)$. Then, to calculate the median, we solve the following:

(A14) $$\int_{0}^{t}(n+1)\left(n+2\right)p^{n}\left(1-p\right)dp=0.5$$

and the confidence interval by solving

(A15) $$\begin{array}{ll} & \;\;\int_{0}^{t}(n+1)\left(n+2\right)p^{n}\left(1-p\right)dp=0.05\\ \mathrm{and} & \\ & \;\;\int_{0}^{t}(n+1)\left(n+2\right)p^{n}\left(1-p\right)dp=0.95. \end{array}$$

All three integrals require approximation methods to solve.

**Example** **A3.** *9 positive and 1 negative*.

From the above, we must solve the following.

(A16) $$\int_{0}^{t}(n)\left(n+1\right)p^{n-1}\left(1-p\right)dp=\alpha$$

By integration, we have the following equation.

(A17) $$\left(n+1\right)t^{n}-nt^{n+1}=\alpha$$

Thus, in our case, we have the following.

(A18) $$11t^{10}-10t^{11}=\alpha$$

We begin by letting α=0.5, i.e., finding the median. Usually, one would simply apply Newton’s method and iterate the following:

(A19) $$a_{n+1}=a_{n}-\frac{11*a_{n}^{10}-10*a_{n}^{11}}{110*\left(a_{n}^{9}-a_{n}^{10}\right)}$$

but the catch is that one has to choose $a_{0}$ close to the true root. Because of the exponents, Newton’s method is very sensitive to initial data. A straightforward way to find the solution is to use a method described in Table A1. Note that all we are doing is determining which interval the value lies in and dividing that interval into two equal parts at each step.

**Table A1:** The median for the 9 positive and 1 negative.

1/2	0.005859
½ + ¼ = 3/4	0.197097
3/4/ + 1/8 = 7/8	0.59192
7/8 − 1/16 = 13/16	0.360472
13/16 + 1/32 = 27/32	0.468599
27/32 + 1/64 = 53/64	0.52865
53/64 − 1/128 = 105/128	0.498176
105/128 + 1/256 = 211/256	0.513305
211/256 − 1/512 = 421/512	0.505713

**Example** **A4.**
*The statistics for all draws of 8 subjects.*

**Table A2:** The statistics for drawing 8 subjects.

Pos.	Neg.	Formula	Mean	Median	Mode	90% Interval
8	0	$p^{8}$	0.9	0.920	1	(0.717, 0.994)
7	1	$8p^{7}\left(1-p\right)$	0.8	0.82	0.875	(0.571, 0.959)
6	2	$28p^{6}{\left(1-p\right)}^{2}$	0.7	0.714	0.75	(0.451, 0.902)
5	3	$56p^{5}{\left(1-p\right)}^{3}$	0.6	0.608	0.625	(0.345, 0.832)
4	4	$70p^{4}{\left(1-p\right)}^{4}$	0.5	0.5	0.5	(0.252, 0.749)
3	5	$56p^{3}{\left(1-p\right)}^{5}$	0.4	0.394	0.375	(0.169, 0.656)
2	6	$28p^{2}{\left(1-p\right)}^{6}$	0.3	0.287	0.25	(0.098, 0.550)
1	7	$8p{\left(1-p\right)}^{7}$	0.2	0.180	0.125	(0.042, 0.430)
0	8	${\left(1-p\right)}^{8}$	0.1	0.080	0	(0.006, 0.283)
